# Efficacy of Levetiracetam Use in Neonatal Seizure: A Retrospective Cohort Study

**DOI:** 10.3390/neurosci7010008

**Published:** 2026-01-12

**Authors:** Faisal Aqeel Alsehli, Jahad Alghamdi, Abdulaziz Homedi, Saif Alsaif, Kamal Ali, Wed S. Alzahrani, Nataleen A. Albekairy, Aiman A. Obaidat, Mohammad S. Shawaqfeh, Buthaynah Ahmed Alawad, Atheer Abdulaziz Alfulaij, Norah Mohammed Almamoon, Abdulkareem M. Albekairy

**Affiliations:** 1 Neonatal Intensive Care, King Abdulaziz Medical City, Ministry of National Guard Health Affairs, Riyadh 11481, Saudi Arabia; 2 College of Medicine, King Saud Bin Abdulaziz University for Health Sciences, Riyadh 11481, Saudi Arabia; 3 King Abdullah Bin Abdulaziz International Research Centre, Riyadh 11426, Saudi Arabia; 4 Drug Sector, Saudi Food and Drug Authority, Riyadh 13312, Saudi Arabia; 5 Research Center, King Saud Medical City, Riyadh 11461, Saudi Arabia; 6 College of Pharmacy, King Saud Bin Abdulaziz University for Health Sciences, Riyadh 11481, Saudi Arabia; 7 Specialized Medical Center Hospital, Riyadh 11586, Saudi Arabia; 8 District Sales Manager-Acino, Riyadh 12334, Saudi Arabia; 9 Dallah Hospital, Riyadh 11652, Saudi Arabia

**Keywords:** levetiracetam, neonatal seizure, antiepileptics, seizure freedom

## Abstract

**Highlights:**

- Safety Profile: LEV avoids the risk of neuronal apoptosis and hippocampal transcriptome dysregulation associated with traditional GABAergic agents like phenobarbital.
- Clinical Efficacy: Successful seizure control was achieved in up to 83% of cases when LEV was used either as monotherapy or combined with standard care.
- Electroclinical Dissociation: A critical risk when combining LEV and PB; visible movements may stop while electrical brain activity continues, necessitating the use of cEEG or aEEG for accurate burden assessment.
- Dosing Optimization: Emerging data suggests higher loading doses (up to 60 mg/kg) may be required in complex cases like HIE to reach therapeutic targets.
- Future Directions: Transitioning LEV from a refractory-use medication to a primary agent requires large-scale RCTs focusing on long-term neurodevelopmental outcomes.

**Abstract:**

Neonatal seizures are common complications in neonatal intensive care units. They have been noticed to be more common in preterm infants, but they can also affect term infants. Levetiracetam is a broad-spectrum antiepileptic drug that has been studied to manage seizures, yet limited data are available on its use in neonatal seizures. **Objectives:** Study the effect of levetiracetam on neonatal seizures in terms of maintaining seizure freedom after the initiation of levetiracetam and investigating its safety profile in the neonate population. **Method:** Retrospective cohort study comparing two groups of patients identified through accessing their medical profiles after searching the following keywords: phenobarbital, levetiracetam, and neonatal seizures amongst all NICU admissions in King Abdulaziz Medical City, Ministry of National Guard Health Affairs, from the period between December 2016 and January 2020. Forty-eight patients were included based on the inclusion/exclusion criteria. The selected sample was further subclassified into 28 neonates who received phenobarbital and 20 who received levetiracetam. **Results:** Seizure control was significantly observed in neonates with onset <24 h and those born at <37 weeks GA. In the first arm, 22 out of 28 neonates achieved seizure freedom while using phenobarbital; in the second arm, 11 out of 20 neonates achieved seizure control on levetiracetam after failing with phenobarbital. While seizure control was better achieved by phenobarbital, it was found that almost 57% of the first arm developed side effects on phenobarbital; however, only 10% of the neonates on levetiracetam developed side effects. While PB remains effective for acute suppression, LEV demonstrated a superior safety profile with no serious adverse events and a high rate of successful seizure management as an add-on therapy (83% control in combined cohorts). **Conclusions:** The study concluded that using levetiracetam could result in improved outcomes. LEV is a safe and effective alternative or adjunct to PB. Its use may mitigate the neurotoxic risks associated with GABAergic drugs, though continuous EEG monitoring is essential to ensure electrical seizure cessation and avoid electroclinical dissociation. The number of patients who received levetiracetam initially is not considered a representative sample to reach a conclusion on the use of levetiracetam as an effective monotherapy.

## 1. Introduction

Neonatal seizures are common complications in neonatal intensive care units (NICUs). They occur more commonly in preterm neonates yet they also occur in full-term neonates; therefore, they require strict guidelines for their management [1]. This is particularly critical because the neonatal brain is in a state of inherent hyperexcitability, where excitatory glutamatergic systems develop more rapidly than inhibitory GABAergic pathways. A neonatal electrographic seizure is defined as a sudden, repetitive, evolving, and stereotyped event of abnormal electrographic patterns with an amplitude of at least 2 mV and a minimum duration of 10 s [2]. Without continuous monitoring, clinicians may miss “subclinical” seizures or “electro-clinical dissociation”, where physical movements stop but the brain continues to seize—a phenomenon often exacerbated by the administration of certain antiseizure medications [3]. Neonatal seizures often require an electroencephalogram (EEG) for diagnosis and monitoring [2]. Seizures affect 1–3 neonates per 1000 live births; the prevalence is much higher in premature neonates (10–130/1000 live births) [4]. Diagnosis and classification should follow standardized terminology to ensure consistency across clinical settings [5]. They can be caused by many factors, including vascular, metabolic, infectious, malformation, epileptic syndrome, or be of unknown origin [6]. Seizures in neonates are usually managed using phenobarbital (PB), phenytoin, levetiracetam (LEV), and benzodiazepines (BZDs).

LEV is a broad-spectrum antiepileptic drug (AED) that was approved by the US Food and Drug Administration in 1999 and has become one of the most widely prescribed drugs for the treatment of partial and generalized epilepsy [7]. Its pharmacokinetic profile is ideal for the NICU due to low protein binding and minimal hepatic metabolism. LEV controls seizures through multiple mechanisms and has much less drug–drug interactions based on its pharmacokinetic properties. It was found that LEV is effective in seizure control through affecting GABA turnover in the striatum and decreasing levels of the amino acid taurine. In addition, LEV removed the Zn2C-induced suppression of GABA A-mediated presynaptic inhibition [7]. Synaptic vesicle protein 2 (SV2) is a 12 trans-membrane integral protein present at all synaptic sites. It is not considered essential for synaptic transmission, but SV2A knockout mice exhibit seizures, and thus it is considered target for LEV binding [7]. By binding to SV2A, LEV modulates the release of neurotransmitters during high-frequency neuronal firing without disrupting normal synaptic communication [8]. It is also important to note that LEV has a good safety profile in terms of side effects, making it highly effective and safe to use in the neonatal population. When comparing LEV with PB, many studies have shown that fewer cognitive and motor impairments are seen at 24 months in the LEV group when compared with the PB group [1]. In a randomized controlled study that was conducted over 18 months from a level III NICU, 100 full-term and preterm neonates with clinical seizures were included [9]. Patients were assigned randomly to receive either PB or LEV and the study concluded that LEV was safer, more effective, and has a promising good outcome in treating neonatal seizures compared to PB [6]. Another clinical trial included 245 neonates with seizures, and they were managed by intravenous PB. Intravenous phenytoin was added if PB was not effective, and if refractory seizures were observed, oral LEV was added [10]. This perspective is further supported by Loiacono et al. (2016), who reported a 100% response rate when LEV was utilized as a first-line treatment, highlighting its potential to replace more sedating alternatives [11]. The study concluded that either oral or intravenous LEV is effective in controlling neonatal seizures, and in emergency conditions, its use as a first line of therapy is more effective than PB without any significant side effects [10]. A retrospective study reviewed 37 preterm infants who were treated with LEV as the first-line antiepileptic [8]. The study reported that 21 neonates were seizure-free when LEV was administered at the end of the first week, and no additional antiepileptic drug was required to control their seizures, while 16 neonates needed additional antiepileptic drugs during LEV treatment [12]. A prospective feasibility study included neonates presenting with clinical seizures. The study included a total of 38 newborns with EEG-confirmed seizures, considering pathologic EEG findings [13]. They were treated with LEV as a first-line AED, which revealed that 30 neonates were seizure-free at the end of the first week. A total of 27 neonates remained seizure-free at four weeks and EEGs markedly improved in 25 patients at four weeks, with excellent tolerability profiles for all the patients [13]. The specific aim of our study is to assess the efficacy and safety of LEV in the management of neonatal seizures as a mono- or add-on therapy in comparison with standard management using PB. Given the known heterogeneity in retrospective cohorts, we aim to provide a nuanced analysis that accounts for clinical and EEG characteristics to ensure a more homogeneous comparison of treatment outcomes.

## 2. Method

In this study, we retrospectively scanned patients through accessing their medical profiles on the Best Care system in King Abdulaziz Medical City (KAMC) Ministry of National Guard-Health Affairs, Kingdom of Saudi Arabia, through searching for the following keywords: phenobarbital, levetiracetam, and neonatal seizures. Patients were screened over the period between December 2016 and January 2020. We also considered patients if they had seizure onset at an age between 0 and 28 days of life. They were included if they met the following criteria: (1) Presented with seizures. (2) Received LEV to manage their seizure. We excluded neonates if they had (1) seizures caused by hypoglycaemia; (2) seizures caused by electrolyte imbalance (hypomagnesemia, hypocalcaemia, and hyponatremia); (3) and those whose seizures were responsive to pyridoxine. To facilitate a structured comparison, patients were categorized based on their pharmacological response: the “PB-Responsive” group (Standard Management) and the “LEV-Intervention” group (Sequential Management). We identified 48 neonates (30 males and 18 females) in total. Of these, 28 patients achieved seizure freedom solely on the standard PB protocol, while 20 patients required the transition to LEV after PB failed to provide adequate control. Neonatal seizures were identified through clinical manifestations (eye movement, rolling up or staring, and smacking, abnormal movement of the upper or lower extremities) neurological and physical examinations, and electroencephalography (EEG) findings. EEG records were scrutinized for specific patterns, including spike-and-wave discharges or burst-suppression, to ensure that electrographic seizure cessation matched clinical observations. We defined patients as controlled if they maintained seizure freedom status for more than 5 days on antiepileptic medications. The primary endpoint of this study was defined as maintaining seizure freedom on antiepileptic medications. Secondary endpoints were seizure frequency and the number of status epilepticus cases. This study was approved by the Institutional Review Board (IRB) of the King Abdullah International Medical Research Centre (KAIMRC) (RC20/464/R).

### 2.1. Statistical Analysis

All data are expressed as median [interquartile range (IQR)] or number (%). The Kolmogorov–Smirnov and Shapiro–Wilk tests were employed to determine the normality of the data distribution. Data between the groups (Standard PB-only vs. LEV-Intervention) were compared using the Mann–Whitney U-test for continuous variables with non-normal distribution and Fisher’s exact test for categorical outcomes. This non-parametric approach was selected to mitigate the impact of the small, heterogeneous sample sizes inherent in retrospective neonatal data. All reported results are two-sided, and statistical significance was defined as *p* < 0.05. We performed multivariate logistic regression; seizure control was entered as a dependent variable and intervention, gender, gestational age (GA), onset of seizures, and birth weight were independent variables. This model was adjusted to identify whether LEV administration remained a significant predictor of seizure freedom when controlling for baseline clinical disparities. All statistical analyses were conducted using SPSS 23.0 (SPSS, Inc., Chicago, IL, USA).

### 2.2. Results

Between December 2016 and January 2020, a total of 471 medical profiles were screened; 241 patients matched the search keywords from the original population. After applying the inclusion/exclusion criteria to the study population, 48 patients were eligible to be included in the study. The study group was further classified into two arms based on the pharmacological treatment they received during hospitalization. In both arms, neonates age ranged from 0 to 28 days of life, except for three patients in both groups who fell out of this age range due to prolonged hospitalization but had seizure onset within the neonatal period. The neonates’ weight varied between normal birth weight (2.5–3.5 kg), low birth weight < 2.5 kg, very-low birth weight < 1.5 kg, and extremely low birth weight < 1 kg. The neonates’ gestational age (GA) was either preterm < 37-week or term ≥ 37-week. The baseline characteristics of the included patients are listed in Table 1. The first arm consisted of 28 patients (58.3%) who received PB for the initial management of seizures where 22 neonates (78.60%) maintained seizure control and 6 neonates (21.40%) were uncontrolled despite optimum therapeutic management with PB, including those who were transferred prior to maintaining seizure control or died. In 57.10% of the sample (16 neonates), elevation in liver function tests (LFTs) was reported as the main side effect. Of the total first-arm cohort, two patients (7.1%) required the addition of phenytoin for the management of seizures. Neonates were loaded with PB (10–30 mg/kg/day) intravenously then transitioned to a maintenance dose of 2–5 mg/kg/day, with a calculated mean of 4.47 mg/kg/day in two divided doses which maintained seizure freedom.

In the second arm, neonates were loaded with LEV (10–45 mg/kg/day) then given a maintenance dose of 10–80 mg/kg/day, with a mean maintenance of 36 mg/kg/day in two divided doses which maintained seizure control. A comparative analysis of the primary endpoint was conducted to evaluate the clinical efficacy of both protocols. By comparing and evaluating the results from both groups’ outcomes to identify seizure control, a *p* value of 0.117 was obtained, indicating no statistical superiority of LEV over PB in terms of seizure control in this cohort. However, these findings support the role of LEV as a viable add-on therapy after failing standard management with PB.

A significant disparity was observed regarding the safety profiles of the two medications. While there was a high incidence of elevated LFTs (AST and ALT) in the first arm after PB initiation, there was no significant incidence of side effects in the second group (i.e., two patients only), with a *p* value < 0.001, indicating a significantly better safety profile for LEV in the neonate population. Regarding the patients who were on PB, the incidence of side effects was as follows: seven neonates (43.75%) had AST elevation while the remaining neonates (56.25%) had elevation in both AST and ALT. The incidence of this side effect was noticed with the initiation of PB; interestingly, after the maintenance dose was administered, the levels remained above the normal range (>55 U/L).

**Figure 1:**
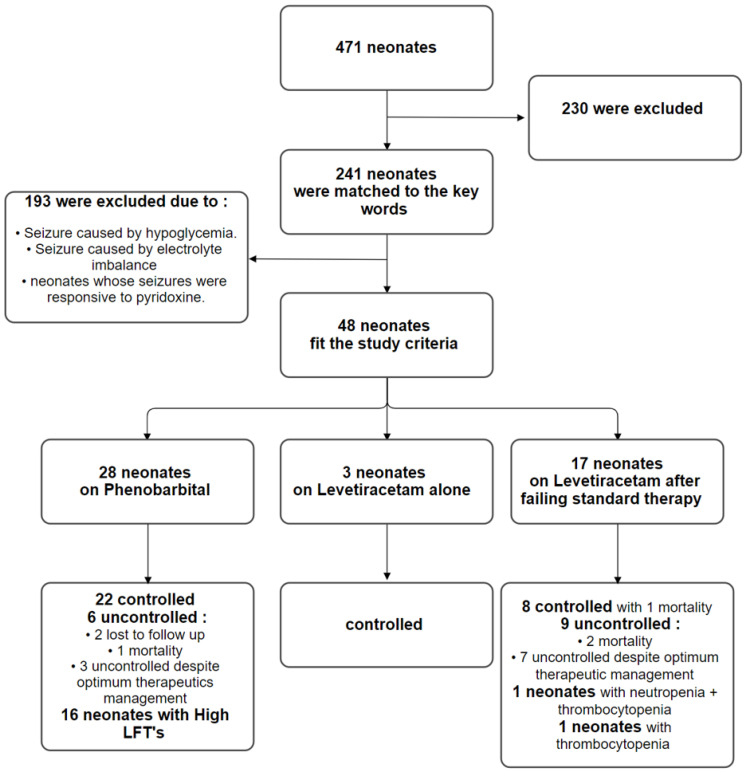
Study Design Flow Chart.

The second arm included 20 patients (41.66%) who received LEV for seizure management after failing standard therapy with PB. In this group, three patients (15%) received LEV as initial management for their seizures. Within this intervention cohort, eleven neonates (55%) were controlled with one incidence of mortality, and nine neonates (45%) were uncontrolled due to optimum therapeutic management or mortality occurring prior to maintaining seizure freedom. Regarding adverse events in the LEV group, two neonates (10%) experienced side effects after receiving LEV, mainly neutropenia and/or thrombocytopenia (Figure 1). Notably, status epilepticus was not reported in either arm during the study period.

We conducted a multivariate analysis to identify the potential association between the baseline characteristics and interventions used in both arms, with seizure control entered as the dependent variable. We found that only male gender was statistically significant [odds ratio: 6.2; 95% (1.2–32.3); *p* = 0.028], indicating that males were more responsive to antiepileptic medications.

## 3. Discussion

Neonatal seizures are a frequent clinical challenge and are often linked to adverse neurodevelopmental outcomes; they are frequently the first indicator of neurological dysfunction. Currently, there is a lack of standardized management protocols, leading to treatment strategies that vary significantly based on individual hospital practices. While recent international guidelines emphasize the need for rapid diagnostic and therapeutic intervention, they acknowledge that high-quality evidence for second-line pharmacotherapy remains scarce [14,15].

Levetiracetam (LEV) is a broad-spectrum antiepileptic drug that is increasingly prescribed in neonatal intensive care units. Its mechanism of action—targeting the synaptic vesicle protein SV2A—differs fundamentally from conventional agents like phenobarbital (PB). This distinction may allow it to bypass the physiological challenges neonates present, such as the reversed chloride gradient in immature neurons that can make GABAergic drugs like PB less effective [16]. Our study hypothesized that LEV could maintain seizure freedom whether utilized as a monotherapy or as an adjunctive therapy to standard approaches.

In our study, neonates were selected regardless of baseline category, age of onset, gestational age (GA), birth weight, or congenital abnormalities. This inclusive criterion supports the generalizability of our findings across diverse neonatal populations [12,17]. This is consistent with recent observational studies that have included both term and preterm neonates to reflect real-world NICU populations [18]. In the first arm (standard therapy), there were two patients who developed seizures eight months after discontinuing PB. On the other hand, there was no incidence of recurrent seizures in the LEV group. Within our study groups, data were missing for only a few subjects: two regarding GA, two for birth weight, and three for age of onset.

According to our results, seizure control was more observed when neonates had seizure onset at less than 24 hrs. of life, male gender, or were born at less than 37-weeks GA. However, this result could not be accurate due to the higher percentage of males in our study sample. These results are consistent with previous results, which proved that LEV could be considered as a good option as an add-on therapy after failing to control seizures with PB, although in our study, the sample was too small to establish its place as first-line management.

Our results observed better seizure control in neonates with an onset within the first 24 h of life, males, and those born at less than 37 weeks GA. The efficacy of LEV has been demonstrated in a study that included a total of 36 patients, in which 17 (47%) patients had seizure control after administering LVE as a monotherapy, and 18 patients were controlled after the administration of LEV in combination with PB or fosphenytoin. In total, 30 (83%) patients achieved seizure control on LEV either as a monotherapy or as an add-on therapy [19]. Similarly, retrospective data from Korean neonatal cohorts showed that 94% of neonates achieved seizure cessation within one week of LEV initiation [20]. In a randomized controlled study conducted on 122 neonates with seizures, 50 neonates received LEV and 60% of them were controlled. In contrast, of the other 50 neonates who received PB, 50% of the neonates were controlled. A total of 3 neonates who failed LEV management responded to PB, while 16 neonates exhibited response to LEV after failing PB [21]. However, a high-impact randomized controlled trial (RCT) found that PB was superior to LEV for achieving 24 h seizure freedom (80% vs. 28%) [22]. This suggests that while LEV is a potent second-line option, its role as a primary agent is still being debated, especially since loading doses of 40–60 mg/kg may be necessary for optimal efficacy [14,22].

After initiating LEV therapy in neonates who were antiepileptic naive or who failed with PB at their seizure onset, no serious adverse events were noticed, although two neonates developed hematologic reactions (thrombocytopenia and neutropenia). Such reactions could be contributed to causes other than LEV administration. These results are consistent with the safety outcomes from other studies, which concluded that LEV could be used effectively without potential side effects [19,23]. Crucially, experimental evidence indicates that while PB exposure can dysregulate the hippocampal transcriptome and induce neuronal apoptosis in the immature brain, LEV does not appear to produce these neurotoxic effects [24,25].

The limitations of our study include the retrospective design, relatively small sample size (48 neonates), and variable follow-up periods amongst the neonates in both arms. The RCT study highlights that phenobarbital demonstrated greater efficacy in seizure cessation but also noted the adverse effects associated with it [22]. Furthermore, the efficacy of LEV as an add-on therapy for the treatment of seizures in neonates was limited and is still controversial; the highest seizure reduction rate was seen after lidocaine administration [26]. Finally, a study similar to ours compared LEV to PB when used as first-line and second-line therapies for seizures in a cohort of 104 neonates. The results indicate similar efficacy and suggested better safety even though both medications were needed for full control [27].

In neonates, a seizure may have both a clinical manifestation (visible jerking and stiffening) and an electrical correlate on an EEG. Dissociation occurs when the clinical symptoms disappear—often due to medication—while the electrical seizure activity in the brain continues unabated. Traditional antiseizure medications (ASMs) like phenobarbital (PB) and phenytoin are more effective at suppressing the motor pathways in the brainstem and spinal cord than they are at stopping electrical discharge in the neonatal cortex. When you add levetiracetam (LEV) to a patient already on PB, the risk of masking ongoing brain activity increases. PB provides significant sedation and muscle relaxation. When combined with LEV, the neonate may become even more “quiet” clinically, making it nearly impossible to detect subtle seizures without continuous EEG (cEEG). Loiacono et al. suggests that the “seizure burden”—the total time the brain spends in a state of electrical seizure—is what correlates with long-term neurodevelopmental outcomes, regardless of whether the baby is physically moving. The future role of levetiracetam (LEV) in neonatology is transitioning from a refractory-use medication to a potential primary agent. As highlighted by Loiacono et al. (2016), the high efficacy (up to 100% response in their cohort) and safety profile of LEV suggest that it could mitigate the neurotoxic risks—specifically neuronal apoptosis—associated with GABAergic drugs like phenobarbital [11]. Future research should prioritize large-scale, randomized controlled trials to determine optimal loading doses and to quantify long-term neurodevelopmental outcomes compared to standard care. Additionally, the integration of continuous vEEG or aEEG will be essential to ensure that LEV achieves true electrical seizure cessation rather than merely suppressing clinical symptoms [11]. The transition from clinical observation to mandatory electrographic monitoring (cEEG or aEEG) is essential to ensure that the cessation of physical movements is not merely a masking effect of polypharmacy. Furthermore, the variability in loading doses observed in this study suggests that standardized, higher-dose protocols may be necessary to achieve optimal therapeutic concentrations [25]. The current literature highlights that up to 40–60% of surviving neonates with high seizure burdens develop long-term sequelae such as cerebral palsy or learning difficulties [15].

## 4. Conclusions

In conclusion, this retrospective study has emphasized the potential benefit of adding LEV to patients’ antiepileptic remedy who failed initial management with PB, with almost no side effects. The use of LEV as a monotherapy is not well studied due to traditional hospital practices in the management of neonatal seizures, which resulted in a very small sample size. Statistically, while traditional agents like PB have long been favored for their rapid clinical suppression of motor symptoms, our analysis aligns with the recent literature suggesting that LEV offers a superior safety profile regarding neurotoxicity and apoptotic risk. Further studies are required to establish a clear result on the potential benefit of using LEV. In addition, a study with a longer follow-up period would indicate the neurodevelopmental outcomes of LEV in the early management of seizures. Ultimately, while PB remains a potent first-line intervention, LEV is emerging as a critical component of the neonatal anticonvulsant toolkit. Future research should prioritize large-scale, randomized controlled trials to determine optimal loading doses and quantify long-term neurodevelopmental outcomes compared to standard care.

## Figures and Tables

**Table 1:** Baseline Characteristics.

	All	Phenobarbital	Levetiracetam
**GA** **^1^** **(week)**	37.0 (30.75–39.0)	38.0 (29.75–40.0)	36.5 (33.0–38.0)
**Birth Weight** **(kg)**	2.53 (1.54–3.14)	3.0 (1.45–3.40)	2.10 (1.62–2.94)
**Gender**	18 females	9 females	9 females
	30 males	19 males	11 males
**Age at Onset** **(week)**	1.5 (0.0–8.25)	1.0 (0.0–8.0)	2.0 (0.0–13.0)
**Congenital Abnormalities**			
**HIE** **^2^**	7	6 (85.7%)	1 (14.28%)
**CHD** **^3^**	10	6 (60%)	4 (40%)
**Prematurity** **^4^**	12	7 (58.33%)	5 (41.6%)

^1^ GA (G=gestational age): the neonates’ gestational age was either preterm < 37-week or term ≥ 37-week. ^2^ HIE (hypoxic–ischemic encephalopathy): is a brain dysfunction that occurs when the brain does not receive enough oxygen or blood flow for a period of time. ^3^ CHD (congenital heart disease): is a problem with the structure of the heart; it is present at birth. ^4^ Prematurity: neonates born at less than 37 weeks’ gestation.

## Data Availability

The data presented in this study are available on request from the corresponding author according to the ethical and legal standards of the healthcare facility.
